# Psychometric refinement and validation of the Entrepreneurial Personality Inventory across distinct occupational groups

**DOI:** 10.3389/fpsyg.2026.1893893

**Published:** 2026-08-17

**Authors:** Mariela Pavalache-Ilie, Ana-Maria Cazan, Camelia Truța, Denisa Elena Tomulescu, Marcela Rodica Luca

**Affiliations:** 1 Faculty of Psychology and Education Sciences, Transilvania University of Brasov, Brașov, Romania; 2 Romanian Academy, Institute of Philosophy and Psychology "Constantin Rădulescu-Motru”, Bucharest, Romania

**Keywords:** confirmatory factor analysis, entrepreneurial intention, entrepreneurial personality, entrepreneurial personality inventory, locus of control, measurement invariance, proactive personality, psychometric validation

## Abstract

**Introduction:**

Entrepreneurial personality is associated with entrepreneurial intention and business-related behavior, yet existing measures remain fragmented and insufficiently validated across occupational groups. The present study aimed to psychometrically refine and validate the Entrepreneurial Personality Inventory (EPI) across distinct occupational groups.

**Methods:**

The study included 897 Romanian participants distributed across four groups: students enrolled in entrepreneurial education programs, matched students, entrepreneurs, and employees. The initial inventory included 113 items assessing seven dimensions of entrepreneurial personality. Exploratory and confirmatory factor analyses were used to test the factorial structure of the instrument, while preliminary evidence of convergent validity and measurement invariance across groups were also examined.

**Results:**

Exploratory factor analysis supported a six-factor solution after the removal of problematic items and an unstable conformity factor. The final dimensions were motivation, persistence, entrepreneurial abilities, social skills, independence, and risk propensity. Confirmatory factor analyses indicated moderate fit indices for the revised model and satisfactory internal consistency coefficients. Entrepreneurial personality dimensions were positively associated with proactive personality and internal locus of control. Significant group differences were observed; however, these findings should be interpreted cautiously because only configural measurement invariance was supported across occupational groups.

**Discussion:**

The findings provide preliminary psychometric support for a revised six-factor structure of the Entrepreneurial Personality Inventory and support its use for research purposes. Differences between groups suggest potential distinctions between entrepreneurial intention and entrepreneurial practice, emphasizing the importance of validating entrepreneurial personality measures across diverse populations.

## 1 Introduction

Entrepreneurial personality has long been associated with a broad range of entrepreneurial outcomes, including entrepreneurial intentions, entrepreneurial behavior, venture creation, and, in some studies, business performance (Kerr et al., 2018; Rauch and Frese, 2007). Over the past decades, entrepreneurship research has progressively shifted from viewing entrepreneurial behavior as exclusively determined by economic or contextual variables toward more integrated psychological models. In this context, personality-related variables have become increasingly important for understanding why some individuals are more likely to identify opportunities, initiate ventures, and persist despite obstacles and uncertainty. However, despite the growing interest in entrepreneurial personality, the development of comprehensive and psychometrically sound instruments remains limited.

Research in the field consistently suggests that individuals who decide to engage in entrepreneurial activities display specific personality characteristics, including achievement motivation, risk propensity, tolerance for uncertainty and stress, innovativeness, need for autonomy, and proactive personality (Rauch and Frese, 2007). Previous studies show that entrepreneurs differ from non-entrepreneurs on several dispositional characteristics, including proactive personality, internal locus of control, need for achievement, openness to experience, and entrepreneurial self-efficacy (Leutner et al., 2014; Obschonka and Stuetzer, 2017). Also, empirical evidence consistently supports the fact that personality-related variables explain why some individuals are more likely than others to initiate entrepreneurial activities, identify business opportunities, and persist in uncertain and competitive environments (Brandstätter, 2011). While traditional approaches have focused on the Big Five personality model, recent literature suggests that studying and understanding entrepreneurial personality should take into consideration more specific mechanisms that mediate the relation with entrepreneurial behaviors (Obschonka and Stuetzer, 2017).

Despite the growing body of literature, the conceptualization and measurement of entrepreneurial personality remain somewhat fragmented. In some studies, isolated traits are measured (i.e., entrepreneurial self-efficacy), while in others, narrow combinations of characteristics are examined without consistent operationalization. Furthermore, many instruments often remain limited to specific groups, lacking validation across diverse occupational categories, which leads to heterogeneous findings across studies (Howard, 2023). Much of the literature relies on student samples or individuals involved in entrepreneurial education programs, while comparatively fewer studies directly compare these groups with entrepreneurs and non-entrepreneurial employees.

These limitations highlight the need for multidimensional instruments capable of assessing entrepreneurial personality across distinct occupational groups while demonstrating adequate psychometric properties. The present study aimed to psychometrically refine and validate the Entrepreneurial Personality Inventory (EPI), an existing multidimensional measure designed to assess personality characteristics associated with entrepreneurial behavior. Specifically, the study examined the factorial structure, internal consistency, preliminary evidence of convergent validity, and measurement invariance of the instrument across students enrolled in entrepreneurial education programs, matched students, entrepreneurs, and employees. By assessing validity across diverse samples, the present research aims to provide a robust tool capable of identifying entrepreneurial potential, applicable across distinct occupational contexts.

## 2 Entrepreneurial personality as a multidimensional construct

Research on entrepreneurial personality increasingly supports the view that entrepreneurship cannot be adequately explained through a single dispositional characteristic. Rather, entrepreneurial functioning appears to emerge from the interaction of multiple motivational, cognitive, behavioral, and interpersonal dimensions that collectively facilitate entrepreneurial engagement and adaptation to uncertain environments (Obschonka and Stuetzer, 2017).

Early research in entrepreneurship primarily relied on broad personality frameworks such as the Five-Factor Model (FFM). Meta-analytic evidence indicates that entrepreneurs generally report higher levels of extraversion, conscientiousness, and openness to experience, alongside lower levels of neuroticism and agreeableness, compared to managers or non-entrepreneurs (Brandstätter, 2011; Zhao and Seibert, 2006), even though effect sizes for each personality dimension are relatively small. This could suggest that broad personality dimensions may not adequately capture the specific psychological processes underlying entrepreneurial behavior. Consequently, recent studies have investigated entrepreneurial characteristics that are conceptually closer to entrepreneurial activities and decision-making processes, including self-efficacy, proactivity, autonomy, achievement motivation, and risk propensity (Leutner et al., 2014; Rauch and Frese, 2007).

The entrepreneurial personality concept evolved from the static trait-based approach to more dynamic and integrative perspectives. For example, Obschonka and Stuetzer (2017) conceptualize entrepreneurial personality not as an isolated set of traits, but as a complex psychological architecture integrating both stable dispositional tendencies and more adaptive personality characteristics. Within this framework of Entrepreneurial Personality System (EPS), broad personality traits such as extraversion or conscientiousness constitute a relatively stable dispositional core, while entrepreneurial adaptations - including entrepreneurial self-efficacy, passion, and risk orientation - are considered more malleable and susceptible to change through learning processes and interactions with the environment (Obschonka and Stuetzer, 2017). This distinction is particularly important because it suggests that entrepreneurial personality reflects not only individual differences but also adaptive psychological processes shaped by entrepreneurial experiences and contextual influences across time.

Among the most studied dimensions of entrepreneurial personality are achievement motivation, proactivity, self-efficacy, autonomy, and risk propensity. Achievement motivation refers to the tendency to pursue excellence, accomplish challenging goals, and persist despite difficulties (McClelland, 1965). Studies show that entrepreneurs score significantly higher on achievement motivation than managers or non-entrepreneurs, particularly entrepreneurs oriented toward innovation and business growth (Brandstätter, 2011; Stewart and Roth, 2007). Need for achievement is also strongly correlated with entrepreneurial engagement and entrepreneurial success (Kerr et al., 2018).

Several studies also highlight the role of entrepreneurial self-efficacy and internal locus of control in entrepreneurial engagement and persistence (Kerr et al., 2018; Newman et al., 2019). Individuals with an internal locus of control tend to perceive outcomes as dependent on their own actions and decisions, whereas entrepreneurial self-efficacy reflects confidence in one’s ability to successfully manage entrepreneurial tasks and challenges. Higher levels of entrepreneurial self-efficacy are associated with entrepreneurial intention, opportunity recognition, venture creation, and persistence in the face of obstacles (Newman et al., 2019). These findings suggest that individuals who believe they can influence outcomes and effectively handle entrepreneurial demands are more likely to engage in entrepreneurial activities and sustain entrepreneurial efforts over time.

Similarly, risk propensity seems to be more strongly associated with entrepreneurial intention than with long-term entrepreneurial performance, as successful entrepreneurship also requires strategic decision-making to face financial uncertainty, incomplete information, and unpredictable outcomes (Brandstätter, 2011). Meta-analyses suggest that entrepreneurs do not necessarily seek risk for its own sake, but rather possess a different perception of it, being able to function optimally under conditions of uncertainty (Frese and Gielnik, 2014).

Proactivity and innovativeness are also frequently studied in entrepreneurial research and included in measures of entrepreneurial personality. Proactive individuals tend to anticipate opportunities, initiate change, and actively shape their environment rather than merely reacting to external demands (Bolton and Lane, 2012). Innovativeness involves openness to experimentation, creativity, and the generation of novel solutions, characteristics frequently associated with opportunity recognition and venture development (Howard, 2023).

In recent years, several integrative frameworks have been developed that conceptualize entrepreneurial personality as a dynamic system. Obschonka and Stuetzer (2017) propose the Entrepreneurial Personality System (EPS), which integrates broad personality traits, entrepreneurial characteristic adaptations, self-concepts, and contextual influences into a coherent framework. According to this perspective, stable personality dispositions interact continuously with environmental experiences and entrepreneurial activities across the lifespan. Entrepreneurial personality cannot, therefore, be reduced to a set of personality traits; its analysis should also take into consideration the fact that entrepreneurial experiences shape entrepreneurial behavior.

Similarly, Howard and Boudreaux (2024), through a systematic literature review and meta-analysis, identified seven dimensions most consistently associated with entrepreneurial personality: innovativeness, risk-taking propensity, achievement orientation, proactiveness, locus of control, self-efficacy, and autonomy orientation. Their findings supported the existence of a higher-order entrepreneurial personality factor underlying these dimensions and demonstrated consistent relationships with entrepreneurial attitudes, intentions, status, and performance outcomes.

Overall, the cited empirical studies and meta-analysis support the conceptualization of entrepreneurial personality as a multidimensional construct integrating motivational, cognitive, behavioral, and interpersonal characteristics. Such multidimensional approaches appear more effective in explaining entrepreneurial outcomes than personality traits (Leutner et al., 2014). Therefore, the development of comprehensive multidimensional measures remains an important objective in entrepreneurship research.

## 3 Theoretical foundations of the entrepreneurial personality dimensions

### 3.1 Motivation

The motivation dimension of the Entrepreneurial Personality Inventory is primarily grounded in the literature on achievement motivation, which has long been regarded as one of the central psychological characteristics underlying entrepreneurial behavior. McClelland (1965) proposed that individuals with a strong need for achievement are more likely to pursue challenging goals, assume personal responsibility for outcomes, and seek feedback regarding their performance. These characteristics are relevant in entrepreneurial contexts, where individuals must define their own performance standards, tolerate uncertainty, and remain committed to long-term goals despite frequent setbacks (Rauch and Frese, 2007).

Within the EPI, the Motivation dimension reflects individuals’ tendency to pursue challenging goals, strive for high levels of performance, and remain strongly oriented toward personal achievement.

### 3.2 Persistence

Persistence is widely regarded as a key characteristic of entrepreneurial behavior because entrepreneurial activity rarely follows a linear trajectory and often requires individuals to sustain effort despite uncertainty, repeated failures, and delayed rewards. Consequently, persistence reflects not only determination but also the capacity to maintain goal-directed behavior over extended periods of time. Although closely related to achievement motivation, persistence represents a distinct aspect of entrepreneurial functioning by emphasizing sustained effort in the face of obstacles rather than the desire to achieve challenging goals (Kerr et al., 2018).

Within the EPI, the Persistence dimension reflects individuals’ willingness to maintain effort, complete demanding tasks, and continue pursuing their goals despite setbacks. In the context of measuring entrepreneurial personality, there is clear distinction between motivation and persistence, as separate but strongly related dimensions of entrepreneurial personality.

### 3.3 Entrepreneurial abilities

Entrepreneurial abilities refer to the perceived capacity to recognize opportunities, generate and develop business ideas, mobilize resources, and initiate entrepreneurial action. Rather than reflecting formal business knowledge or technical expertise, this dimension captures a set of self-perceived competencies that enable individuals to identify and exploit entrepreneurial opportunities. Research on entrepreneurial competencies suggests that entrepreneurial activity relies not only on stable personality characteristics but also on competencies that enable individuals to recognize opportunities, mobilize resources, and initiate entrepreneurial action (Bird, 2019; Mitchelmore and Rowley, 2010). Bird (2019) described entrepreneurial competencies as the underlying characteristics that enable individuals to achieve effective entrepreneurial performance, whereas research argued that the recognition and exploitation of entrepreneurial opportunities represent defining elements of the entrepreneurial process (Shane and Venkataraman, 2000). Similarly, Baron (2006) proposed that opportunity recognition relies on cognitive processes that allow entrepreneurs to identify meaningful patterns in complex and uncertain environments.

Within the EPI, Entrepreneurial Abilities represent individuals’ perceived capability to recognize business opportunities, generate entrepreneurial initiatives, and engage in behaviors that support venture creation and development. The present study focuses on the entrepreneurial initiative and opportunity recognition rather than technical business expertise, reflecting the behavioral competencies that facilitate entrepreneurial action.

### 3.4 Social skills

Social skills refer to the interpersonal abilities that enable individuals to establish, maintain, and effectively manage relationships with others. In entrepreneurial contexts, social skills facilitate networking, communication, persuasion, and the mobilization of social resources, all of which are essential for recognizing opportunities, acquiring resources, and developing entrepreneurial ventures. Entrepreneurs’ social competence plays an important role in entrepreneurial activity by facilitating interactions with investors, customers, employees, and business partners (Baron and Markman, 2003). Accordingly, entrepreneurial activity depends not only on technical knowledge or business expertise but also on the ability to build productive interpersonal relationships and gain the support of others (Rauch and Frese, 2007).

Within the EPI, the Social Skills dimension reflects individuals’ perceived ability to establish interpersonal relationships, communicate effectively, and interact confidently in professional and entrepreneurial settings. In the context of measuring entrepreneurial personality, communication skills, interpersonal effectiveness, persuasion, and the ability to establish and maintain professional relationships represent important social resources for entrepreneurial activity.

### 3.5 Independence

Independence reflects the preference for autonomy, self-direction, and personal responsibility in pursuing professional goals. Individuals high in independence prefer to make their own decisions, assume responsibility for their actions, and maintain control over their work rather than relying on external direction.

The need for autonomy has long been recognized as an important motivational characteristic of entrepreneurs. Entrepreneurship offers individuals the opportunity to pursue self-directed goals, exercise discretion in decision making, and create their own work environment, making autonomy one of the central motives underlying entrepreneurial activity (Rauch and Frese, 2007; Shane et al., 2003).

Within the EPI, the Independence dimension reflects individuals’ preference for autonomous action, freedom in decision making, and self-directed work. The present study emphasizes personal autonomy and independence in professional contexts rather than social withdrawal or individualism.

### 3.6 Risk propensity

Risk propensity is widely recognized as one of the core characteristics associated with entrepreneurial behavior, reflecting individuals’ willingness to make decisions under conditions of uncertainty (Rauch and Frese, 2007; Stewart and Roth, 2001). Entrepreneurial activity inherently involves uncertain outcomes related to market acceptance, financial performance, and business survival, making the willingness to accept calculated risks an important component of entrepreneurial action.

Research has consistently shown that entrepreneurs tend to display higher levels of risk propensity than managers and other occupational groups (Stewart and Roth, 2001). However, entrepreneurial risk propensity should not be equated with impulsive or reckless behavior. Rather, entrepreneurial decision making typically involves evaluating uncertain opportunities, balancing potential gains against possible losses, and making informed decisions despite incomplete information (Forlani and Mullins, 2000; Kerr et al., 2018).

In the context of measuring entrepreneurial personality, Risk Propensity reflects individuals’ willingness to accept uncertainty and engage in calculated risk taking when pursuing entrepreneurial opportunities, focusing on attitudes toward entrepreneurial risk and decision making under uncertainty rather than on general sensation seeking or impulsivity.

## 4 Challenges in measuring entrepreneurial personality

Despite the substantial growth of research on entrepreneurial personality, its measurement presents several challenges. One difficulty arises from the lack of consensus regarding the dimensions of entrepreneurial personality. Existing studies operationalize entrepreneurial personality using different combinations of traits, including achievement motivation, proactivity, innovativeness, autonomy, self-efficacy, locus of control, and risk-taking propensity, for example (Howard, 2023). Consequently, findings across studies are often difficult to compare.

A related challenge concerns the diversity of existing measurement approaches. Early instruments frequently focused on isolated entrepreneurial characteristics or broad dispositional models such as the Five-Factor Model, which may not fully capture the specific psychological processes involved in entrepreneurial behavior (Leutner et al., 2014) or the broader psychological profile associated with entrepreneurship (Cuesta et al., 2018). More recent measures have adopted multidimensional approaches. For example, Bolton and Lane (2012) developed the Individual Entrepreneurial Orientation (IEO) scale, conceptualizing entrepreneurial orientation through innovativeness, proactiveness, and risk-taking propensity. Cuesta et al. (2018) developed the Battery for the Assessment of the Enterprising Personality (BEPE), which assesses eight dimensions of entrepreneurial personality, including self-efficacy, autonomy, achievement motivation, internal locus of control, optimism, stress tolerance, innovativeness, and risk-taking. More recently, Howard (2023) proposed the Entrepreneurial Personality Scale (EPS), integrating seven dimensions consistently identified in the literature: innovativeness, risk-taking propensity, achievement orientation, proactiveness, locus of control, self-efficacy, and autonomy orientation.

Existing measures differ considerably in the dimensions included and in the theoretical conceptualization of entrepreneurial personality, leading to partial conceptual overlap (Howard, 2023). Some instruments emphasize entrepreneurial orientation and behavioral tendencies, whereas others focus more heavily on motivational or dispositional characteristics. As Obschonka and Stuetzer (2017) distinguish, the nature of entrepreneurial personality itself is not necessarily relatively stable; it also consists of more malleable characteristic adaptations shaped through entrepreneurial experience and contextual influences. However, many existing measures assess entrepreneurial characteristics as equally stable traits, without adequately accounting for the developmental and context-dependent aspects of entrepreneurial functioning. For example, while conscientiousness might be a stable trait, entrepreneurial self-efficacy is a state-like construct that can be developed through education and experience (Obschonka and Stuetzer, 2017).

Another challenge concerns the cross-group validity of existing instruments. Many entrepreneurial personality measures were developed using relatively homogeneous samples, particularly university students or individuals participating in entrepreneurial education programs. Although these populations are highly relevant for studying entrepreneurial intention, they may not adequately represent the psychological characteristics associated with sustained entrepreneurial activity and business experience. For example, proactivity and risk-taking propensity may be particularly relevant during the early stages of entrepreneurial intention formation, whereas persistence, adaptive decision-making, and interpersonal competencies may become increasingly important during long-term entrepreneurial engagement (Brandstätter, 2011). Consequently, evidence regarding factorial stability, measurement invariance, and construct validity across entrepreneurs, employees, and non-entrepreneurial populations remains limited.

Taken together, these challenges highlight the need for multidimensional entrepreneurial personality instruments that are theoretically integrated, psychometrically robust, and applicable across different occupational contexts. Developing measures that demonstrate factorial stability and validity among students, entrepreneurs, and employees may contribute to a more comprehensive understanding of entrepreneurial personality and how it translates into business behavior.

## 5 The present study

The present study aimed to develop and validate a multidimensional Entrepreneurial Personality Inventory (EPI) designed to assess personality characteristics. Based on previous research regarding entrepreneurial personality and entrepreneurial behavior, the inventory was conceptualized as a multidimensional measure including motivational, behavioral, interpersonal, and autonomy-related characteristics associated with entrepreneurial activity. Although the initial version of the Entrepreneurial Personality Inventory was organized into seven theoretically derived dimensions, the present validation study aimed to identify the latent structure that best represented the empirical relationships among the items. Consequently, the final six dimensions represent a psychometrically supported conceptualization of entrepreneurial personality while remaining grounded in the broader theoretical framework that guided the original development of the inventory.

The study examined the factorial structure of the inventory through exploratory and confirmatory factor analyses and evaluated its internal consistency and preliminary evidence of convergent validity in relation to proactive personality and locus of control dimensions. In addition, the stability of the factorial structure across distinct occupational groups was investigated through multi-group confirmatory factor analysis.

A further objective of the study was to examine potential differences between entrepreneurial intention and entrepreneurial practice by comparing entrepreneurial education students, matched students, entrepreneurs, and employees on entrepreneurial personality dimensions. Based on previous research, it was expected that entrepreneurial personality dimensions would show positive associations with proactive personality and internal locus of control and would differ across occupational groups.

## 6 Methods

### 6.1 Participants

The study included 897 Romanian participants distributed across four occupational groups. The first group consisted of 216 university students enrolled in an entrepreneurial education program designed to develop entrepreneurial competencies. A demographically comparable group of 216 university students who were not involved in entrepreneurial education activities was also included. The two student groups were comparable in terms of age, gender, and educational background. The third group comprised 250 entrepreneurs who owned or managed an active business at the time of data collection. Participants were recruited through entrepreneurial associations, professional business networks, and continuing education programs for entrepreneurs. The sample included entrepreneurs from a variety of economic sectors, including services, trade, and small and medium-sized enterprises. The overall sample included 487 men and 410 women. Detailed demographic characteristics of the participants are presented in Table 1.

**Table 1:** Demographic characteristics of the participants across the four groups.

Group	Men	Women	*N*	*M _Age_*	*SD*
Entrepreneurial education students	105	111	216	24.57	5.19
Matched students	105	111	216	24.65	5.18
Entrepreneurs	191	59	250	43.59	10.08
Employees	86	129	215	37.01	9.59
Total	487	410	897	–	–

### 6.2 Procedure

Data were collected from participants recruited from universities, entrepreneurial training programs, private companies, and entrepreneurial organizations in Romania. Participation was voluntary and anonymous. All participants completed the questionnaires individually after providing informed consent. The instruments were administered in paper-and-pencil format.

The study was approved by the Faculty Council (Faculty of Psychology and Educational Sciences, Transilvania University of Brașov, Approval No. 98/29.09.2021, and was conducted in accordance with the ethical principles outlined in the Declaration of Helsinki.

### 6.3 Measures

Three self-report instruments were administered in the present study: the Entrepreneurial Personality Inventory, the Multidimensional Locus of Control Scale, and the Proactive Personality Scale.

Entrepreneurial Personality was assessed using The Entrepreneurial Personality Inventory (EPI) (Luca and Cazan, 2011). EPI was developed to assess personality characteristics associated with entrepreneurial behavior and entrepreneurial intention. The development of the inventory was based on an extensive review of the literature concerning entrepreneurial personality, entrepreneurial competencies, and psychological characteristics associated with entrepreneurial behavior Item generation was guided by theoretical models emphasizing motivational, interpersonal, behavioral, and autonomy-related dimensions of entrepreneurial functioning. The initial item pool was developed to capture a broad range of characteristics frequently associated with entrepreneurial activity, including achievement motivation, persistence, social skills, creativity, independence, risk propensity, and resource organization. Items were formulated to reflect both entrepreneurial intention and entrepreneurial behavior in occupational contexts. Prior to data collection, the items were reviewed for clarity, redundancy, and conceptual relevance. The initial version of the inventory consisted of 113 items organized into seven theoretically derived dimensions: risk propensity (21 items), social skills (14 items), entrepreneurial abilities (17 items), creativity (17 items), independence (17 items), achievement motivation (17 items), and resource organization (10 items). Participants responded using a 5-point Likert scale ranging from 1 (strongly disagree) to 5 (strongly agree). The psychometric properties and factorial structure of the inventory were examined in the present study through exploratory and confirmatory factor analyses. The complete English translation of the original item pool and a detailed summary of the psychometric refinement process are provided in Supplementary Appendix A.

Locus of Control was measured with The Multidimensional Locus of Control Scale (Levenson, 1981), a self-report instrument measuring individuals’ beliefs regarding the extent to which life outcomes are determined by internal control, powerful others, or chance. Participants responded using a 5-point Likert scale ranging from 1 (strongly disagree) to 5 (strongly agree). The scale includes three dimensions: Internality, Powerful Others, and Chance. In the current sample, Cronbach’s alpha coefficients were 0.73 for Internality, 0.79 for Powerful Others, and 0.75 for Chance.

Proactive personality was assessed using the Proactive Personality Scale (Bateman and Crant, 1993), which measures individuals’ tendency to initiate change, identify opportunities, and actively influence their environment. The instrument consists of 17 items organized into a single dimension. Participants responded using a 5-point Likert scale ranging from 1 (strongly disagree) to 5 (strongly agree). In the current sample, the scale demonstrated good internal consistency (*α* = 0.91).

### 6.4 Data analysis

Data analyses were conducted using SPSS and AMOS. The factorial structure of the Entrepreneurial Personality Inventory was examined through exploratory factor analysis (EFA) and confirmatory factor analysis (CFA). An exploratory dimensionality analysis based on Principal Component Analysis (PCA) with Varimax rotation was initially conducted to examine the underlying structure of the inventory and identify poorly performing items. The resulting factor structure was subsequently evaluated using confirmatory factor analysis. The exploratory analysis was conducted primarily to identify poorly performing items and reduce the initial item pool prior to confirmatory testing of the resulting measurement model.

Confirmatory factor analyses were subsequently performed to evaluate the fit of the factorial models identified through EFA. Model fit was assessed using multiple fit indices, including the chi-square to degrees of freedom ratio (χ^2^/df), the Comparative Fit Index (CFI), the Tucker–Lewis Index (TLI), and the Root Mean Square Error of Approximation (RMSEA). Following commonly accepted recommendations (Hu and Bentler, 1999; Kline, 2011), values below 0.08 for RMSEA and values approaching 0.90 for CFI and TLI were considered indicative of acceptable model fit. Mahalanobis distance showed no evidence of multivariate outliers.

Measurement invariance across occupational groups was examined using multi-group confirmatory factor analysis (MGCFA). Configural and metric invariance models were compared using chi-square difference tests and changes in fit indices.

Internal consistency was evaluated using Cronbach’s alpha, McDonald’s omega, and composite reliability (CR) coefficients. Pearson correlation coefficients were computed to examine convergent validity associations between entrepreneurial personality dimensions, proactive personality, and locus of control dimensions. Group differences were examined using analyses of covariance (ANCOVA), with occupational group as the fixed factor, age entered as a covariate, and gender included as an additional fixed factor.

## 7 Results

### 7.1 Exploratory factor analysis

An exploratory factor analysis was conducted to examine the factorial structure of the Entrepreneurial Personality Inventory and to identify problematic items. The exploratory item refinement was conducted iteratively. At each stage, items with factor loadings below 0.30, substantial cross-loadings, or conceptual inconsistency were removed before repeating the analysis. Overall, 41 items were removed during the exploratory phase, resulting in a 72-item solution. A summary of the item refinement process, the final retained items organized by factor, and the items excluded during psychometric refinement are provided in Supplementary Tables A1–A3. These items were removed from subsequent analyses. The revised exploratory factor analysis supported a seven-factor structure consisting of 72 items, explaining 47.75% of the total variance. The identified factors were labeled Motivation, Persistence, Entrepreneurial Abilities, Social Skills, Independence, Risk Propensity, and Conformity. Internal consistency coefficients ranged from 0.50 to 0.92 across the seven dimensions. Because the Conformity factor demonstrated low internal consistency and conceptual instability, subsequent analyses focused on a more parsimonious six-factor solution. The factorial structure, explained variance, and reliability coefficients for the retained factors are presented in Table 2.

**Table 2:** Factor loadings, explained variance, and reliability coefficients for the Entrepreneurial Personality Inventory.

Item	Motivation	Persistence	Entrepreneurial abilities	Social skills	Independence	Risk propensity	Conformity
EP17	0.652						
EP29	0.648	0.303					
EP21	0.630						
EP14	0.629						
EP22	0.617						
EP15	0.615						
EP24	0.576						
EP12	0.569					0.323	
EP20	0.549				0.330		
EP30	0.529						
EP57	0.528	0.432					
EP7	0.524	0.497					
EP19	0.524		0.396				
EP63	0.488			0.372			
EP41	0.480			0.419			
EP58	0.470	0.411					
EP56	0.430						
EP9	0.382						
EP36	0.363						
EP97	0.302	0.724					
EP107		0.660					
EP91	0.402	0.599					
EP85	0.387	0.560					
EP79	0.455	0.557					
EP109		0.535	0.320				
EP100		0.499		0.328			
EP95		0.495		0.306			
EP110	0.339	0.495					
EP99		0.483		0.364			
EP65	0.401	0.481		0.333			
EP112		0.440	0.319				
EP98		0.438			0.322	0.360	
EP111		0.435					
EP49	0.302	0.420	0.380				
EP25			0.736				
EP18			0.680				
EP26			0.613				
EP47			0.600				
EP103		0.343	0.559				
EP82			0.542	0.326			
EP27			0.537				
EP33			0.526	0.307			
EP75			0.500	0.314			
EP10			0.466				
EP104			0.433				
EP67				0.754			
EP68				0.625			
EP66				0.571			
EP39				0.556			
EP69	0.302			0.554			
EP4	0.315			0.469			
EP32				0.460			
EP44			0.373	0.418			0.315
EP70					0.670		
EP101					0.620		
EP96		0.322			0.611		
EP105					0.595		
EP106			0.314		0.550		
EP77	0.302				0.480		
EP28					0.406		
EP52			0.425			0.621	
EP59			0.329			0.615	
EP1						0.607	
EP92			0.301			0.576	
EP45			0.408			0.555	
EP23			0.338			0.435	
EP84							0.555
EP64							0.541
EP13							0.493
EP5							0.488
EP6							−0.458
EP93							−0.352
Eigenvalues	8.29	6.75	5.96	4.81	3.43	3.21	1.90
% of variance	11.51	9.38	8.28	6.68	4.77	4.46	2.64
Cronbach’s α	0.92	0.92	0.86	0.84	0.73	0.82	0.50
Number of items	19	15	11	8	7	6	6

Extraction method: Principal Component Analysis. Rotation method: Varimax with Kaiser normalization. Factor loadings below 0.30 are not displayed.

### 7.2 Confirmatory factor analysis

Confirmatory factor analyses (CFA) were conducted to evaluate the factorial structure identified through the exploratory factor analysis. Several alternative models were tested, including seven-factor and six-factor solutions with and without correlated error terms. The seven-factor model with correlated errors demonstrated improved fit indices compared to the model without correlated errors. However, negative error variances associated with several items loading on the Conformity factor suggested instability of this dimension, consistent with its low internal consistency. Correlated residuals were introduced only between items belonging to the same latent construct when the item content or wording suggested shared variance beyond the common latent factor. No correlated residuals were specified across different constructs. A revised six-factor model excluding the Conformity dimension was tested.

The six-factor model with correlated errors demonstrated improved and relatively moderate fit indices. Model evaluation was based on commonly recommended criteria for structural equation modeling (Hu and Bentler, 1999; Kline, 2011). Mahalanobis distance analyses indicated no evidence of multivariate outliers.

To further improve model fit, items with low factor loadings (< 0.45), substantial cross-loadings, or conceptual inconsistency were removed from the model. Specifically, items EP9 and EP36 showed the lowest loadings on the Motivation factor, while item EP20, despite its acceptable loading, was conceptually inconsistent with the construct of motivation. Similarly, items EP10 and EP28 demonstrated weak loadings on their intended factors, whereas item EP18 referred to more specific entrepreneurial behaviors compared to the remaining items of the Entrepreneurial Abilities dimension.

The removal of items EP20, EP9, EP36, EP18, EP10, and EP28 improved model fit without reducing the internal consistency coefficients of the retained dimensions. The final six-factor solution demonstrated the best overall fit among the tested models (Table 3). The final retained items and the excluded items are documented in Supplementary Tables A2, A3.

**Table 3:** Fit indices for the confirmatory factor analysis models of the Entrepreneurial Personality Inventory.

Model	χ^2^/df	CFI	TLI	AIC	RMSEA (90% CI)
Seven-factor model without correlated errors	10735.99/2463***	0.727	0.717	11065.99	0.061 (0.060–0.062)
Seven-factor model with correlated errors	8725.61/2425***	0.801	0.791	8725.61	0.054 (0.053–0.055)
Six-factor model without correlated errors	9262.43/2064***	0.752	0.743	9262.43	0.061 (0.060–0.064)
Six-factor model with correlated errors	7357.56/2030***	0.817	0.806	7357.56	0.054 (0.052–0.055)
Final six-factor model with item removal	6108.49/1663***	0.836	0.825	6442.49	0.055 (0.053–0.056)

CFI = Comparative Fit Index; TLI = Tucker–Lewis Index; AIC = Akaike Information Criterion; RMSEA = Root Mean Square Error of Approximation; CI = confidence interval. ***p < 0.001.

The reliability of the final six-factor solution was further evaluated using Cronbach’s alpha, McDonald’s omega, and composite reliability coefficients (Table 4). Cronbach’s alpha coefficients ranged from 0.73 to 0.92, McDonald’s omega coefficients ranged from 0.73 to 0.92, and composite reliability coefficients ranged from 0.72 to 0.92, indicating satisfactory internal consistency across all retained dimensions.

**Table 4:** Reliability estimates for the final six-factor model.

Dimension	Cronbach’s α	McDonald’s *ω*	Composite reliability
Motivation	0.916	0.917	0.915
Persistence	0.920	0.920	0.916
Entrepreneurial abilities	0.859	0.861	0.849
Social skills	0.839	0.839	0.837
Independence	0.736	0.737	0.817
Risk propensity	0.819	0.820	0.721

#### 7.2.1 Sensitivity analysis

To evaluate the robustness of the retained factor structure, an additional sensitivity analysis was conducted following the reviewer’s methodological recommendations. The total sample was randomly divided into independent development and validation subsamples. Exploratory factor analysis was performed on the development sample using Principal Axis Factoring with Direct Oblimin rotation, and the resulting solution was subsequently evaluated through confirmatory factor analysis in the independent validation sample. Although the alternative solution reproduced the general conceptual organization of the EPI, it did not demonstrate superior model fit compared with the final model retained in the present study. Therefore, the original six-factor solution was retained. Detailed results are presented in Supplementary Appendix B.

### 7.3 Confirmatory factor analysis across occupational groups

The final six-factor model was subsequently tested separately across the four occupational groups included in the study: students enrolled in entrepreneurial education programs, matched students, entrepreneurs, and employees. The best model fit was observed for students enrolled in entrepreneurial education programs, whereas less satisfactory fit indices emerged for the remaining groups, particularly for entrepreneurs (Table 5). These findings suggest that the proposed factorial structure may be more strongly associated with entrepreneurial intention and entrepreneurial training contexts than with entrepreneurial practice itself.

**Table 5:** Fit indices for the six-factor model across occupational groups.

Group	χ^2^/df	CFI	TLI	AIC	RMSEA (90% CI)
Entrepreneurial education students	2877.99/1689***	0.716	0.702	3159.98	0.057 (0.054–0.060)
Matched students	3606.83/1680***	0.693	0.676	3906.82	0.073 (0.070–0.076)
Entrepreneurs	4284.13/1664***	0.736	0.719	4616.13	0.079 (0.077–0.081)
Employees	3234.34/1687 ***	0.732	0.719	3520.34	0.065 (0.062–0.069)

CFI = Comparative Fit Index; TLI = Tucker–Lewis Index; AIC = Akaike Information Criterion; RMSEA = Root Mean Square Error of Approximation; CI = confidence interval. ***p < 0.001.

To further examine the stability of the factorial structure across groups, multi-group confirmatory factor analysis (MGCFA) was conducted. The configural invariance model demonstrated moderate fit indices, indicating a similar general factorial structure across groups. However, comparisons between the configural and metric invariance models revealed a significant deterioration in model fit, supporting a lack of metric invariance across occupational groups.

Inspection of factor loadings across groups indicated lower factor loadings among entrepreneurs compared to the remaining groups. Partial metric invariance models also failed to demonstrate invariance of factor loadings for any of the six factors. These findings indicate that the six-factor structure was reproduced across occupational groups with varying levels of model fit. Because measurement invariance analyses supported configural but not metric invariance, these results should be interpreted as preliminary evidence regarding the applicability of the EPI across occupational contexts rather than as definitive evidence of structural equivalence (Table 6). Therefore, subsequent comparisons between occupational groups should be interpreted cautiously, as differences may partly reflect variation in measurement properties rather than only true differences in the underlying constructs.

**Table 6:** Multi-group confirmatory factor analysis of the Entrepreneurial Personality Inventory.

Model	χ^2^/df	TLI	CFI	RMSEA	Δχ^2^	Δdf	AIC
Configural invariance	14037.36/6652***	0.718	0.699	0.035	–	–	15373.35
Metric invariance	14781.89/6836***	0.696	0.685	0.036	744.53***	184	15749.89

CFI = Comparative Fit Index; TLI = Tucker–Lewis Index; AIC = Akaike Information Criterion; RMSEA = Root Mean Square Error of Approximation; CI = confidence interval. ***p < 0.001.

### 7.4 Convergent validity

Pearson correlation analyses were conducted to examine associations between the dimensions of the Entrepreneurial Personality Inventory, proactive personality, and locus of control dimensions. As expected, all entrepreneurial personality dimensions were positively associated with proactive personality and internal locus of control. Weak negative correlations were identified between entrepreneurial personality dimensions and external locus of control dimensions, particularly Chance and Powerful Others (Table 7). These findings provide preliminary evidence for the convergent validity of the Entrepreneurial Personality Inventory, as the observed associations were consistent with theoretically expected relationships with proactive personality and locus of control.

**Table 7:** Pearson correlations between entrepreneurial personality dimensions, proactive personality, and locus of control.

Variable	1	2	3	4	5	6	7	8	9	10
1. Motivation	–	0.796**	0.495**	0.661**	0.334**	0.396**	0.651**	0.605**	−0.173**	−0.200**
2. Persistence		–	0.577**	0.698**	0.436**	0.446**	0.733**	0.638**	−0.120**	−0.189**
3. Entrepreneurial abilities			–	0.616**	0.427**	0.657**	0.595**	0.363**	0.032	−0.019
4. Social skills				–	0.322**	0.503**	0.633**	0.539**	−0.068*	−0.147**
5. Independence					–	0.348**	0.378**	0.287**	0.171**	0.118**
6. Risk propensity						–	0.481**	0.263**	0.058	−0.038
7. Proactive personality							–	0.624**	−0.062	−0.132**
8. Internality								–	0.079*	0.042
9. Chance									–	0.767**
10. Powerful others										–

N = 897. *p < 0.05. **p < 0.01.

### 7.5 Group differences

Differences between occupational groups were examined using analyses of covariance (ANCOVA), with age entered as a covariate and gender included as an additional fixed factor. Descriptive statistics and ANCOVA results are presented in Table 8. The overall pattern of group differences remained significant after controlling for these demographic variables. Significant group differences emerged for all entrepreneurial personality dimensions except Independence. Students enrolled in entrepreneurial education programs consistently reported the highest scores across most entrepreneurial personality dimensions, proactive personality, and internal locus of control. In contrast, entrepreneurs generally reported lower scores on several entrepreneurial personality dimensions compared to students. Entrepreneurs reported higher scores on external locus of control dimensions, particularly Chance and Powerful Others, compared to the remaining groups. Effect sizes ranged from small to large across the analyzed dimensions.

**Table 8:** Descriptive statistics and ANCOVA results for occupational group differences across entrepreneurial personality dimensions, proactive personality, and locus of control.

Variable	Group	*M*	SD	*F*	*p*	Partial η^2^
Motivation	Entrepreneurial education students	4.39	0.31	86.01	<0.001	0.227
	Matched students	4.10	0.48			
	Entrepreneurs	3.35	0.72			
	Employees	4.00	0.49			
Persistence	Entrepreneurial education students	4.20	0.39	64.26	<0.001	0.180
	Matched students	3.83	0.50			
	Entrepreneurs	3.32	0.73			
	Employees	3.81	0.60			
Entrepreneurial abilities	Entrepreneurial education students	3.68	0.53	43.21	<0.001	0.129
	Matched students	2.98	0.70			
	Entrepreneurs	3.27	0.73			
	Employees	3.12	0.75			
Social skills	Entrepreneurial education students	3.96	0.47	27.37	<0.001	0.086
	Matched students	3.59	0.65			
	Entrepreneurs	3.24	0.74			
	Employees	3.58	0.60			
Independence	Entrepreneurial education students	3.29	0.63	1.19	0.314	0.004
	Matched students	3.30	0.64			
	Entrepreneurs	3.27	0.75			
	Employees	3.36	0.66			
Risk propensity	Entrepreneurial education students	3.29	0.63	9.99	<0.001	0.033
	Matched students	2.91	0.78			
	Entrepreneurs	3.18	0.72			
	Employees	3.11	0.80			
Proactive personality	Entrepreneurial education students	3.90	0.41	32.61	<0.001	0.100
	Matched students	3.52	0.50			
	Entrepreneurs	3.24	0.76			
	Employees	3.52	0.59			
Internality	Entrepreneurial education students	3.86	0.38	37.60	<0.001	0.114
	Matched students	3.71	0.47			
	Entrepreneurs	3.11	0.80			
	Employees	3.57	0.56			
Chance	Entrepreneurial education students	2.46	0.52	16.05	<0.001	0.052
	Matched students	2.68	0.53			
	Entrepreneurs	2.89	0.76			
	Employees	2.59	0.59			
Powerful others	Entrepreneurial education students	2.39	0.54	15.19	<0.001	0.049
	Matched students	2.60	0.57			
	Entrepreneurs	2.87	0.79			
	Employees	2.55	0.67			

Entrepreneurial education students (*n* = 216), matched students (*n* = 216), entrepreneurs (*n* = 250), and employees (*n* = 215). Means (M) and standard deviations (SD) are presented for each group. Group differences were examined using analyses of covariance (ANCOVA), with occupational group as the fixed factor, age entered as a covariate, and gender included as an additional fixed factor. Partial η^2^ is reported as the measure of effect size.

## 8 Discussion

The present study examined the psychometric properties and factorial structure of the Entrepreneurial Personality Inventory (EPI) in a Romanian sample across multiple occupational groups. Overall, the findings support a multidimensional conceptualization of entrepreneurial personality and provide preliminary psychometric evidence for the revised six-factor structure of the Entrepreneurial Personality Inventory. The results should be interpreted as supporting the psychometric refinement of the instrument rather than as definitive validation of a finalized measure.

The additional split-sample sensitivity analysis based on Principal Axis Factoring further supported the overall conceptual organization of the inventory but did not provide evidence for a psychometrically superior measurement model (see Supplementary Appendix B). These findings increase confidence that the retained six-factor solution is reasonably robust across alternative exploratory strategies while also highlighting areas requiring further refinement.

The exploratory and confirmatory analyses supported a six-factor solution including Motivation, Persistence, Entrepreneurial Abilities, Social Skills, Independence, and Risk Propensity. These findings are consistent with multidimensional models of entrepreneurship suggesting that entrepreneurial personality should not be conceptualized as a single global trait, but rather as a configuration of motivational, interpersonal, behavioral, and self-regulatory characteristics that support entrepreneurial functioning (Cuesta et al., 2018; Howard, 2023). Similar to previous instruments such as the BEPE battery (Cuesta et al., 2018) and the Entrepreneurial Personality Scale (Howard, 2023), the present results suggest that entrepreneurial personality cannot be adequately reduced to isolated traits or broad personality dimensions alone. The results contribute to the growing literature suggesting that entrepreneurial personality is best conceptualized as a complex and multidimensional construct rather than a single dispositional characteristic (Howard and Boudreaux, 2024; Obschonka and Stuetzer, 2017).

The initial seven-factor model included a Conformity dimension; however, this factor exhibited low internal consistency, conceptual instability, and problematic parameter estimates in confirmatory analyses. Negative error variances and unstable item functioning suggested that conformity-related characteristics may not represent a coherent entrepreneurial dimension within the present context. Given that entrepreneurship is frequently associated with autonomy, innovation, independence, and nonconformity, the weak coherence of this factor appears theoretically plausible. Consequently, the retention of a more parsimonious six-factor solution appears both psychometrically and conceptually justified. Among the retained dimensions, Motivation and Persistence demonstrated the highest factor loadings and reliability coefficients within the final model. This finding aligns with previous entrepreneurship literature emphasizing achievement motivation, goal orientation, perseverance, and sustained effort as central characteristics of entrepreneurial engagement and long-term business activity (Brandstätter, 2011; Stewart and Roth, 2007). More specifically, persistence, sustained goal pursuit, initiative, and motivational engagement may constitute core psychological mechanisms underlying entrepreneurial behavior. Entrepreneurship frequently involves prolonged uncertainty, repeated setbacks, and high personal responsibility, making motivational resilience particularly relevant for entrepreneurial functioning. The strong representation of these dimensions within the EPI may therefore reflect the central role of motivational processes in sustaining entrepreneurial behavior over time.

The other retained factors–Entrepreneurial Abilities, Social Skills, and Risk Propensity–show consistency with recent theoretical frameworks of entrepreneurial personality, suggesting that entrepreneurial functioning reflects the integration of multiple adaptive competencies rather than isolated dispositional tendencies. Previous research has shown that entrepreneurial activity requires not only motivation and initiative but also interpersonal adaptability, opportunity recognition, networking abilities, and tolerance for uncertainty (Leutner et al., 2014; Rauch and Frese, 2007). The inclusion of these dimensions within the EPI is also consistent with the Entrepreneurial Personality System framework proposed by Obschonka and Stuetzer (2017).

The present findings should be viewed as an important step in the ongoing psychometric evaluation of the EPI, while further validation in independent samples and cultural contexts remains necessary. Although these modifications improved model fit, the need to eliminate multiple items suggests that some items may not fully capture the intended constructs within the Romanian cultural context. Certain removed items demonstrated low factor loadings, whereas others appeared conceptually inconsistent with the broader dimensions they were intended to measure. These findings underline the importance of cultural specificity, semantic equivalence, and contextual adaptation in the validation of entrepreneurial personality measures. Given the complexity and breadth of the construct, the obtained fit indices may also reflect the multidimensional and context-sensitive nature of entrepreneurial personality rather than a strictly homogeneous latent structure. The substantial correlations between dimensions may also support the existence of a broader higher-order entrepreneurial personality construct, consistent with recent integrative models (Howard and Boudreaux, 2024).

The convergent validity analyses further supported the construct validity of the EPI. As expected, all entrepreneurial personality dimensions were positively associated with proactive personality and internal locus of control. These findings are highly consistent with previous research showing that entrepreneurial individuals tend to report stronger initiative, active environmental engagement, and greater perceptions of personal control over outcomes (Kerr et al., 2018; Newman et al., 2019). Proactive personality was associated with Motivation and Persistence, suggesting that entrepreneurial personality may be closely linked to active goal pursuit and behavioral self-initiation.

Similarly, the positive associations between EPI factors and internal locus of control are consistent with longstanding findings in the literature (Brandstätter, 2011; Rauch and Frese, 2007). Individuals with stronger internal control beliefs are more likely to perceive entrepreneurial outcomes as contingent upon their own actions, which may facilitate entrepreneurial engagement and persistence despite uncertainty. Together, these findings suggest that the EPI captures psychological tendencies theoretically associated with entrepreneurial initiative, agency, and self-directed behavior.

Overall, the convergent validity findings suggest that the EPI captures an active orientation toward initiative, persistence, personal control, and goal-directed behavior. At the same time, the moderate rather than very high correlations with proactive personality and locus of control further suggest that the EPI captures related but conceptually distinct constructs, providing only indirect evidence regarding discriminant validity and should therefore be interpreted cautiously. Nevertheless, future studies should examine discriminant validity using additional psychometric approaches, such as the Fornell-Larcker criterion or the heterotrait-monotrait ratio (HTMT).

An important contribution of the present study concerns the examination of entrepreneurial personality across occupational groups. The best model fit was identified among students enrolled in entrepreneurial education programs, whereas weaker fit indices emerged among entrepreneurs and employees. Moreover, multi-group confirmatory factor analyses failed to support metric invariance across groups. The lack of metric invariance suggests that the construct may function differently across occupational categories and that particular entrepreneurial characteristics may carry different psychological meanings for students, entrepreneurs, and employees. In other words, similar questionnaire items may carry different psychological meanings for students, entrepreneurs, and employees. Taken together, these findings suggest that the expression and measurement of entrepreneurial personality may vary across occupational contexts. Consequently, the present results should be interpreted as preliminary evidence regarding the applicability of the EPI across different occupational groups rather than as definitive evidence of full cross-group validation.

Although students enrolled in entrepreneurial education programs obtained higher observed scores on several EPI dimensions, these differences should be interpreted cautiously because only configural measurement invariance was supported. Consequently, these findings cannot be interpreted as definitive evidence of latent psychological differences between occupational groups. These differences remained statistically significant after controlling for age and gender, suggesting that they cannot be attributed solely to demographic differences between the occupational groups. Interestingly, entrepreneurs reported lower scores on several entrepreneurial personality dimensions compared to students enrolled in entrepreneurial education programs.

One possible explanation is that entrepreneurial personality may be expressed differently during entrepreneurial intention and entrepreneurial practice. However, because the present study did not assess factors such as entrepreneurial experience, venture characteristics, or occupational demands, these explanations remain speculative. Previous studies have suggested that entrepreneurial intention and entrepreneurial behavior may involve partially distinct psychological processes (Brandstätter, 2011; Obschonka and Stuetzer, 2017). However, the present data do not allow direct conclusions regarding these mechanisms. Moreover, the absence of metric invariance indicates that differences in observed scores may also reflect differences in the measurement properties of the instrument across occupational groups. Therefore, these findings should be interpreted as preliminary and require replication using invariant measurement models and richer occupational data.

The higher levels of external locus of control observed among entrepreneurs may also reflect the realities of entrepreneurial environments. Entrepreneurs operate within dynamic and uncertain economic contexts in which market conditions, financial constraints, and social networks can substantially influence entrepreneurial outcomes. Consequently, individuals engaged in entrepreneurial activity may be more aware of the role that contextual and uncontrollable factors play in shaping business-related outcomes, consistent with contemporary perspectives emphasizing the interaction between individual characteristics and environmental conditions in entrepreneurship (Frese and Gielnik, 2014).

These findings are particularly important because many entrepreneurial personality measures are developed primarily using student populations and are subsequently generalized to entrepreneurial populations without sufficient examination of cross-group validity. The present results suggest that entrepreneurial personality may be partially context-dependent and influenced by entrepreneurial experience and specific demands of the entrepreneurial environment. This interpretation is compatible with dynamic models such as the Entrepreneurial Personality System framework, which emphasizes reciprocal interactions between personality, entrepreneurial activities, and contextual influences across time (Obschonka and Stuetzer, 2017). The present findings provide encouraging evidence for the multidimensional structure and preliminary psychometric properties of the EPI. At the same time, the results indicate that further research is needed to examine the stability of the factorial structure across different populations, establish additional sources of validity evidence, and evaluate the predictive utility of the instrument in relation to entrepreneurial outcomes.

## 9 Limitations and future directions

Several limitations should be considered when interpreting the findings of the present study. First, the reliance on self-report measures may have increased the risk of social desirability effects, self-perception biases, and common method variance. Second, the study was conducted within a Romanian cultural context, which may limit the generalizability of the findings to other cultural or economic environments. The cultural specificity of entrepreneurial attitudes and behaviors may partially explain the instability of certain dimensions and the need for item modifications.

Third, although the final six-factor model demonstrated moderate fit indices, several fit indicators remained within moderate rather than excellent ranges, suggesting that the factorial structure may still require refinement. The elimination of multiple items and the inclusion of correlated error terms further indicate the need for continued psychometric optimization of the instrument. In addition, future studies should further examine the latent structure using alternative extraction and rotation approaches. The exploratory analyses were conducted using principal component analysis, consistent with the analytical strategy adopted during the original development of the instrument. Future studies may benefit from re-examining the factorial structure using alternative extraction methods, such as principal axis factoring or maximum likelihood estimation, together with parallel analysis.

Additionally, the cross-sectional design of the study limits conclusions regarding the temporal stability and developmental dynamics of entrepreneurial personality. No longitudinal data were available to examine whether entrepreneurial personality characteristics remain stable over time or change as a function of entrepreneurial experience and occupational transitions.

Another limitation refers to the entrepreneurs sample. Although entrepreneurs represented diverse business sectors, detailed information regarding entrepreneurial experience (e.g., years of entrepreneurial activity, venture stage, business size, or entrepreneurial history) was not systematically collected. Future studies should examine whether these characteristics influence the structure or expression of entrepreneurial personality. In addition, although the sample size was large, the factorial structure was not cross-validated in an independent sample. To address this limitation, we conducted an additional split-sample sensitivity analysis using Principal Axis Factoring and independent confirmatory factor analysis (Supplementary material S5). Although the alternative solution was conceptually comparable, it did not improve model fit relative to the retained six-factor model. In addition, confirmatory factor analyses were estimated using maximum likelihood estimation. Although this approach is commonly applied to five-point Likert scales in large samples, future validation studies should examine the measurement model using estimators specifically designed for ordinal indicators, such as WLSMV or DWLS.

Furthermore, criterion-related validity was not directly evaluated because no behavioral entrepreneurial outcomes (e.g., venture creation, entrepreneurial self-efficacy, or business performance) were available. Future studies should examine these relationships together with formal tests of discriminant validity such as the Fornell-Larcker criterion and HTMT.

Future research should further investigate the factorial stability of the EPI in larger and more diverse samples and across different cultural contexts. Longitudinal studies may clarify whether entrepreneurial personality characteristics differ between entrepreneurial intention, entrepreneurial engagement, and long-term entrepreneurial maintenance. Additional research should also examine whether the observed lack of metric invariance reflects genuine contextual variability in entrepreneurial personality or limitations associated with item functioning across occupational groups. The lack of metric invariance also limits the direct comparability of latent factor scores across occupational groups and should therefore be considered when interpreting the observed group differences. Refinement and reformulation of problematic items may further improve the psychometric properties of the inventory. Consequently, although the observed differences are informative, they should not be interpreted as definitive evidence of latent mean differences across occupational groups. Rather, they should be viewed as preliminary findings requiring replication using measurement models that demonstrate stronger levels of invariance.

## 10 Implications

Despite these limitations, the present study provides preliminary evidence supporting the usefulness of the revised six-factor version of the Entrepreneurial Personality Inventory in a Romanian context. The EPI may represent a valuable instrument for entrepreneurial education programs, career counseling, entrepreneurial training, and research focused on entrepreneurial personality and entrepreneurial intention.

More specifically, the instrument may help identify psychological characteristics associated with entrepreneurial engagement, persistence, initiative, and risk orientation. Within entrepreneurial education and training contexts, the EPI may support the development of targeted interventions aimed at strengthening entrepreneurial competencies and self-regulatory capacities. In career counseling settings, the instrument may contribute to vocational exploration and the identification of entrepreneurial potential. Furthermore, the inventory may serve as a useful research tool for investigating the multidimensional and context-sensitive nature of entrepreneurial personality across educational, occupational, and cultural environments.

The additional split-sample sensitivity analyses yielded conceptually comparable results but did not improve the psychometric performance of the measurement model, supporting retention of the final six-factor solution while highlighting the need for continued refinement.

Overall, the findings contribute to the growing literature suggesting that entrepreneurial personality is a multidimensional and context-dependent construct whose expression may vary substantially across entrepreneurial intention, entrepreneurial education, and entrepreneurial practice contexts.

## 11 Conclusion

The present study provides preliminary evidence supporting the psychometric validity and multidimensional structure of the Entrepreneurial Personality Inventory (EPI) within a Romanian context. The findings support a revised six-factor model including Motivation, Persistence, Entrepreneurial Abilities, Social Skills, Independence, and Risk Propensity as relevant dimensions associated with entrepreneurial functioning.

The results further suggest that entrepreneurial personality should not be conceptualized as a single global trait, but rather as a multidimensional and context-sensitive configuration of motivational, interpersonal, and behavioral characteristics. In particular, Motivation and Persistence emerged as central dimensions, highlighting the importance of self-regulatory and goal-directed processes in entrepreneurial functioning.

The observed differences across occupational groups should be regarded as preliminary because only configural invariance was supported. Further studies are needed to establish stronger levels of measurement invariance before firm conclusions regarding cross-group differences can be drawn. Lower factor loadings among entrepreneurs further support the view that entrepreneurial functioning is shaped not only by dispositional characteristics, but also by contextual demands, uncertainty, and adaptive experiences associated with real entrepreneurial activity.

Overall, the findings contribute to the growing literature supporting multidimensional approaches to entrepreneurial personality and provide preliminary support for the use of the EPI in entrepreneurial education, counseling, training, and research settings.

## Data Availability

The raw data supporting the conclusions of this article will be made available by the authors, without undue reservation.
