# Supplementary material for: Psychometric refinement and validation of the Entrepreneurial Personality Inventory across distinct occupational groups
Source: Front Psychol. 2026 Aug 17;17:1893893. doi: 10.3389/fpsyg.2026.1893893 (PMC13526577; doi:10.3389/fpsyg.2026.1893893)
Supplement: Supplementary file 1 [file Supplementary_file_1.docx]

Supplementary Material

# Appendix A. English Translation of the Entrepreneurial Personality Inventory Items

This appendix provides the complete English translation of the original 113-item Entrepreneurial Personality Inventory, together with the final retained items and a summary of the item refinement process to facilitate transparency and replication.

1) I enjoy the feeling I experience when taking risks.
2) Others consider me a loner.
3) I am interested in starting my own business.
4) People say that I have a sense of humor.
5) Other people’s opinions are important to me.
6) In high school, I was concerned with obtaining very good grades.
7) I complete projects even when they require a great deal of work.
8) I have taken risks that could have affected my job.
9) People consider me a charming person.
10) My education so far has prepared me to become an entrepreneur.
11) I find new and unusual solutions to everyday problems.
12) You need to take risks in order to succeed.
13) I prefer to work in a traditional organization with a clear hierarchy.
14) I want to achieve important things in life.
15) When it comes to something I truly want, I have a clear image of the desired outcome.
16) I have resigned from a job because I felt frustrated.
17) It is important in life to have close friends.
18) I read publications about business, markets, economics, and management.
19) I view problems as challenges.
20) I usually do things my own way.
21) If I set a goal for myself, I do everything possible to achieve it.
22) I analyze my mistakes in order to learn from them.
23) I am adventurous.
24) For success in business or career, it is important to build a strong social network.
25) I frequently identify business opportunities or ideas with commercial value.
26) I would be willing to risk a large amount of money for a good business opportunity.
27) I have innovative ideas.
28) I tend to rebel against authority.
29) I believe it is important to know what you want in life.
30) In order to learn something new, I often study topics for which I have no formal training.
31) When driving, I pay attention to the speed limit.
32) I enjoy going to parties where I can meet new people.
33) I enjoy developing or building businesses, generating ideas, and turning them into reality.
34) I am adaptable.
35) People say that I am stubborn.
36) I am capable of making small sacrifices in order to obtain long-term rewards.
37) I always keep a notebook or organizer handy to write down my ideas.
38) Seat belts are not necessarily needed in a car.
39) I can easily build relationships with other people.
40) I strive to learn and constantly seek new information that may help me in my business activities.
41) I am curious.
42) I am willing to take moderate risks in order to achieve my goals.
43) I like to take initiative.
44) I am bold.
45) I take risks.
46) I can easily put myself in other people’s situations.
47) I easily identify market opportunities.
48) I tend to be highly intuitive.
49) I enjoy taking responsibility.
50) I have the resources and ability to find solutions to challenges and problems.
51) If you do not have a clear goal in mind, you are wasting your time.
52) Taking risks is as natural to me as breathing.
53) People seem to feel comfortable in my presence.
54) When attending meetings or social events, I usually distribute business cards in order to develop a useful professional network.
55) I can find original uses for common objects.
56) I like to control the way work is carried out.
57) Success motivates me and pushes me toward new achievements.
58) It is important for me to establish both short-term and long-term goals.
59) I enjoy adventure and risk.
60) I see every social situation as an opportunity to expand my social network.
61) I believe that time means money.
62) I am receptive to new ideas.
63) Personal freedom is very important to me.
64) Failure discourages me.
65) When I have something to do, I become fully involved.
66) I take advantage of opportunities.
67) I am good at conversation.
68) It is easy for me to persuade others when arguing a point.
69) I have a rich imagination.
70) I feel a strong need to work independently.
71) Other people’s success motivates me to catch up with them or even surpass them.
72) I never start anything without a clear action plan.
73) I believe that people who take risks reach the top faster than those who do not.
74) Cooperating with others can help me turn my dreams into reality.
75) I frequently interact with others in order to obtain information useful for my business.
76) I experiment with new ways of doing things.
77) I find it annoying to constantly be told what to do.
78) Competition keeps me alive.
79) Once I start a project, I follow it through to the end.
80) I am willing to risk my “skin” for the things I believe in.
81) I feel comfortable in partnerships.
82) I enjoy arranging new business meetings.
83) I am a creative person.
84) I need the approval of my family and friends for every decision I make.
85) I usually do not give up on a project until it is completely finished.
86) I would bet on a good idea even if success were uncertain.
87) I prefer to maintain personal distance from the people I work with.
88) I cultivate relationships with people who I believe may help me in my business or career.
89) I come up with new ideas about how things can be done better and more efficiently.
90) My opinion about myself is more important than other people’s opinions about me.
91) I am persistent.
92) I believe I take more risks than most people.
93) When I have a problem, I immediately think about the people who could help me (friends, family).
94) It is easy for me to sell ideas or products to others.
95) I am capable of finding solutions to challenges and problems.
96) I enjoy working independently.
97) If something needs to be done, I complete it even when it is unpleasant or exhausting.
98) I often follow my instincts.
99) I am a dynamic person who mobilizes others to work.
100) I am capable of adapting quickly and effortlessly to change.
101) It is easier for me to work alone than with others.
102) I see failures as opportunities to learn and develop.
103) I am prepared to take the risks involved in developing a business.
104) I have taken leadership roles.
105) I feel constrained in situations where I must follow strict rules.
106) I would feel suffocated in a position with limited decision-making power.
107) I work as much as necessary to complete a project.
108) If I had to choose, I would rather choose the safe path than the risky one.
109) I feel excited and happy when I can build a business through my work.
110) In my work, I enjoy having varied tasks.
111) I am capable of defending my ideas even when others disagree with me.
112) Challenges and obstacles make me stronger.
113) I sometimes rush headfirst into a business venture without thinking too much about the consequences.

# Supplementary Table A1

| Stage | Number of items |
| --- | --- |
| Initial item pool | 113 |
| Removed after exploratory analysis (low loadings/cross-loadings) | 41 |
| Retained after exploratory analysis | 72 |
| Conformity factor removed | 6 |
| Removed after CFA (EP20, EP9, EP36, EP18, EP10, EP28) | 6 |
| Final instrument | 60 |

# Supplementary Table A2. Final 60-item Entrepreneurial Personality Inventory organized by factor

| **Factor** | **Retained items (EP codes)** | **Number of items** |
| --- | --- | --- |
| Motivation | EP17, EP29, EP21, EP14, EP22, EP15, EP24, EP12, EP30, EP57, EP7, EP19, EP63, EP41, EP58, EP56 | 16 |
| Persistence | EP97, EP107, EP91, EP85, EP79, EP109, EP100, EP95, EP110, EP99, EP65, EP112, EP98, EP111, EP49 | 15 |
| Entrepreneurial Abilities | EP25, EP26, EP47, EP103, EP82, EP27, EP33, EP75, EP104 | 9 |
| Social Skills | EP67, EP68, EP66, EP39, EP69, EP4, EP32, EP44 | 8 |
| Independence | EP70, EP101, EP96, EP105, EP106, EP77 | 6 |
| Risk Propensity | EP52, EP59, EP1, EP92, EP45, EP23 | 6 |

Item wording corresponding to each EP code is provided in Appendix A.

# Supplementary Table A3. Items excluded during psychometric refinement

| **Stage** | **Removed item(s)** | **Primary reason** |
| --- | --- | --- |
| Exploratory refinement | EP2, EP3, EP8, EP11, EP16, EP31, EP34, EP35, EP37, EP38, EP40, EP42, EP43, EP46, EP48, EP50, EP51, EP53, EP54, EP55, EP60, EP61, EP62, EP71, EP72, EP73, EP74, EP76, EP78, EP80, EP81, EP83, EP86, EP87, EP88, EP89, EP90, EP94, EP102, EP108, EP113 | Removed during iterative EFA refinement because of low primary loadings, substantial cross-loadings, and/or conceptual inconsistenc |
| CFA refinement | EP20 | Conceptual inconsistency |
|  | EP9 | Low standardized loading |
|  | EP36 | Low standardized loading |
|  | EP18 | Conceptual inconsistency with the retained factor |
|  | EP10 | Low standardized loading |
|  | EP28 | Low standardized loading |

**Appendix B. Additional Sensitivity Analyses**

To further evaluate the robustness of the retained factor structure, an additional split-sample sensitivity analysis was conducted. The total sample (N = 894) was randomly divided into two independent subsamples (development sample: n = 447; validation sample: n = 447). Exploratory factor analysis was conducted on the development sample using Principal Axis Factoring (PAF) with Direct Oblimin rotation. The number of factors was determined using parallel analysis. The resulting structure was subsequently evaluated through confirmatory factor analysis (CFA) in the independent validation sample using maximum likelihood estimation.

# Supplementary Table B1. Sampling adequacy for the split-sample EFA

| Statistic | Value |
| --- | --- |
| Development sample | 447 |
| Validation sample | 447 |
| KMO | .932 |
| Bartlett's χ² (df = 1128) | 10,550 |
| p | < .001 |

**Confirmatory Factor Analysis**

The factor structure derived from the exploratory analysis was evaluated in the independent validation sample. Two CFA models were examined: Model 1: initial split-sample solution and Model 2: respecified model including theoretically justified correlated residuals within latent dimensions.

**Supplementary Table B2. Fit indices of the split-sample CFA models**

| Fit index | Initial model | Respecified model |
| --- | --- | --- |
| χ² | 3248.11 | 2818.01 |
| df | 1059 | 1049 |
| CFI | .778 | .821 |
| TLI | .764 | .807 |
| RMSEA | .068 | .061 |
| SRMR | .072 | .068 |
| AIC | 54,200 | 53,790 |

Model respecification. Based on the modification indices, a limited number of theoretically justifiable correlated residuals were added between items belonging to the same latent construct. Specifically, residual covariances were specified between EP47–EP48, EP111–EP112, EP101–EP96, EP49–EP50, EP106–EP96, EP52–EP3, EP24–EP25, EP59–EP52, EP106–EP105, and EP91–EP97. These modifications reflected substantial semantic overlap or closely related content and were introduced exclusively within latent dimensions. No correlated residuals were specified between indicators belonging to different factors.

**Supplementary Table B3. Comparison between the sensitivity analysis and the final measurement model**

| Model | CFI | TLI | RMSEA |
| --- | --- | --- | --- |
| Final model retained in manuscript | .836 | .825 | .055 |
| Split-sample sensitivity analysis | .821 | .807 | .061 |

The split-sample analysis generally reproduced the conceptual organization of the inventory obtained in the main analyses. However, the independent CFA based on the PAF-derived solution did not improve global model fit compared with the final model retained in the manuscript. Although the respecified split-sample model showed improved fit relative to the initial CFA (CFI increased from .778 to .821 and RMSEA decreased from .068 to .061), its fit indices remained slightly inferior to those of the final six-factor model reported in the manuscript. Accordingly, these additional analyses were interpreted as a sensitivity analysis demonstrating the robustness of the overall conceptual structure under an alternative extraction method, rather than as evidence supporting replacement of the final measurement model.
